# Repeatability of biometric measures from the IOLMaster 700 in a cataractous population

**DOI:** 10.1371/journal.pone.0297869

**Published:** 2024-02-08

**Authors:** Achim Langenbucher, Nóra Szentmáry, Alan Cayless, Peter Hoffmann, Jascha Wendelstein, David Cooke

**Affiliations:** 1 Department of Experimental Ophthalmology, Saarland University, Homburg/Saar, Germany; 2 Dr. Rolf M. Schwiete Center for Limbal Stem Cell and Aniridia Research, Saarland University, Homburg/Saar, Germany; 3 Department of Ophthalmology, Semmelweis-University, Budapest, Hungary; 4 School of Physical Sciences, The Open University, Milton Keynes, United Kingdom; 5 Augen- und Laserklinik Castrop-Rauxel, Castrop-Rauxel, Germany; 6 Department of Ophthalmology, Johannes Kepler University Linz, Linz, Austria; 7 Great Lakes Eye Care, Saint Joseph, MI, United States of America; 8 Department of Neurology and Ophthalmology, Michigan State University, College of Osteopathic Medicine, East Lansing, MI, United States of America; LV Prasad Eye Institute, INDIA

## Abstract

**Purpose:**

The purpose of this study was to investigate the repeatability of biometric measures and also to assess the interactions between the uncertainties in these measures for use in an error propagation model, using data from a large patient cohort.

**Methods:**

In this cross-sectional non-randomised study we evaluated a dataset containing 3379 IOLMaster 700 biometric measurements taken prior to cataract surgery. Only complete scans with at least 3 successful measurements for each eye performed on the same day were considered. The mean (Mean) and standard deviations (SD) for each sequence of measurements were derived and analysed. Correlations between the uncertainties were assessed using Spearman rank correlations.

**Results:**

In the dataset with 677 eyes matching the inclusion criteria, the within subject standard deviation and repeatability for all parameters match previously published data. The SD of the axial length (AL) increased with the Mean AL, but there was no noticeable dependency of the SD of any of the other parameters on their corresponding Mean value. The SDs of the parameters are not independent of one another, and in particular we observe correlations between those for AL, anterior chamber depth, aqueous depth, lens thickness and corneal thickness.

**Conclusions:**

The SD change over Mean for AL measurement and the correlations between the uncertainties of several biometric parameters mean that a simple Gaussian error propagation model cannot be used to derive the effect of biometric uncertainties on the predicted intraocular lens power and refraction after cataract surgery.

## Background

In cataract surgery, intraocular lens (IOL) power calculation requires reliable biometric measurement data to be used in the calculation concept. As there is no reference measurement for the true biometric data available, absolute accuracy is not a consideration in this context. Systematic measurement errors in the biometric measures (e.g. nonlinearities or offset errors) could be considered with more advanced calculation concepts, but not stochastic variations [1]. Any variation of the biometric measures required for IOL power calculation will directly induce uncertainties in the resulting IOL power (for a preset target refraction) or in the predicted refraction (for a preset IOL power) [1,2]. However, since the stochastic variations of the biometric measures do not combine algebraically, an error propagation concept is required to transform the combined uncertainties of the biometric measures to an uncertainty in the IOL power or the predicted refraction [1–3]. In most cases a Gaussian error propagation model is used as a simplification when estimating the uncertainty of the IOL power or predicted refraction, but this assumes that the effect on the target parameters of the uncertainties in the biometric measures do add arithmetically [3]. Furthermore, Gaussian error propagation assumes that the uncertainties of all biometric measures affecting the target parameter A) are normally distributed, B) are uncorrelated and C) do not show any dependency on the parameter itself. As soon as we notice that at least one of the uncertainties of the biometric measures does not follow a Gaussian distribution, is correlated to another uncertainty of a biometric measure, or shows some heteroscedasticity, the classical method of error propagation does not work properly, and e.g. a Monte-Carlo model is required to transform the uncertainty of the biometric measures to the target parameter [1–3].

In the literature we find several papers which address the uncertainties of biometric measures from modern optical biometers such as the IOLMaster 500 or IOLMaster 700, the LenStar 900, the Pentacam AXL, RevoX, or the OA-2000 [4–14]. In these studies typically a sequence of (e.g. 3) repeat measurements is performed by one rater (intra-rater) or different raters (inter-rater) and the within-subject standard deviation (Sw) of the relevant biometric measures is recorded together with the repeatability, the intra-class correlation (ICC), or the coefficient of variation (CoV) [4–14]. Even if these data are relevant for the clinicians in estimating the uncertainty or repeatability of each biometric measure, they are not sufficient for error propagation as the interaction of the uncertainties in terms of correlations and a potential heteroscedasticity of the uncertainty is not evaluated [3]. As soon as the biometric measures themselves are correlated, we can reasonably expect that the uncertainties will also show some correlation, precluding simple Gaussian error propagation models [1,2].

In the literature we also find some rare papers which use the uncertainties of the biometric measures for error propagation to estimate their effects on the resulting uncertainties of the target parameters IOL power or predicted refraction [3]. However, all of these papers use Gaussian error propagation based on the assumptions A), B), and C) as described earlier. Conditions B) and C) could be tested easily even with a small number of repeat measurements, but condition A) requires a large number of repeat measurements to compare the sequence of measurements from one sample to a normal distribution [3].

As a pre-assumption to establish a proper error propagation model for estimating the effect of uncertainties in the biometric measures on the uncertainty of the predicted IOL power or refraction, the **aim of the present study** was

- to evaluate the within-subject standard deviation Sw of a modern optical biometer together with statistically relevant metrics such as ICC or repeatability for all measures relevant for an IOL power calculation,
- to evaluate the correlations between the uncertainties in the biometric measures, and
- to evaluate the dependency of the uncertainties in the biometric measures on the measures themselves in terms of evaluating heteroscedasticity.

## Methods

### Dataset for our evaluation

A large dataset containing N = 3379 biometric measurements was considered in this study. All measurements were performed at the Great Lakes Eye Care Center (St. Joseph, Michigan, USA) with the IOLMaster 700 (Carl-Zeiss Meditec, Jena, Germany) between March 24, 2021 and July 12, 2022. All procedures performed in studies involving human participants were in accordance with the ethical standards of the Ärztekammer des Saarlandes and with the 1964 Helsinki declaration and its later amendments or comparable ethical standards. The local ethics committee (IRB) has provided a waiver for this study (Ärztekammer des Saarlandes, 157/21), as all data processed in this study were already anonymized at the source before being transferred to us for processing. This precludes any back-tracing of the identity, and therefore informed consent of the patients was not necessary.

This article does not contain any studies with animals performed by any of the authors.

The data were anonymised at source and transferred to a.csv data table using the software module for batch data export. Data tables were reduced to the relevant parameters required for our data analysis, consisting of the following measurements: patient ID, date of birth, examination date, sex (female or male), the laterality (left or right eye), flat (R1a) and steep (R2a) corneal front surface radii of curvature both in mm at axis A1a and A2a in °, flat (R1p) and steep (R2p) corneal back surface radii of curvature both in mm at axis A1p and A2p in °, flat (R1t) and steep (R2t) corneal radii derived from the TK values both in mm at axis A1t and A2t in °, axial length (AL) in mm, central corneal thickness (CCT) in mm, anterior chamber depth (ACD) in mm (measured from corneal epithelium to lens), aqueous depth (AQD) in mm (measured from corneal endothelium to lens), central thickness of the crystalline lens (LT) in mm, horizontal corneal diameter (CD) in mm, pupil size (PUP), and Chang-Waring chord (absolute value CW in mm and orientation CWA in °) [15–18]. The Chang-Waring chord shows the relative position of the Purkinje image I (light reflex originated from the corneal front surface) from the pupil centre.

Only one eye from each subject was included in this study. Where measurements of both eyes were available, one eye was randomly selected. Subjects with missing data or data with a ‘Failed’ or ‘Warning’ in the internal quality check of the IOLMaster 700 for R1a, R2a,A1a, R1p, R2p, A1p, R1t, R2t, A1t, AL, CCT, ACD, AQD, LT, CD, PUP were excluded. Only eyes where a sequence of at least 3 complete measurements was available from the same exam date were considered, and where more than 3 measurements were available, 3 of these were selected randomly and the other measurements discarded. The data were transferred to Matlab (Matlab 2021a, MathWorks, Natick, USA) for further processing.

### Data pre-processing in Matlab

Each patient’s age (Age) in years was derived from the exam date and date of birth. From the corneal front surface data (R1a, R2a, A1a), we extracted the mean keratometric power PKmean = 500·(n_K_-1)·(1/R1a+1/R2a), keratometric astigmatism PKast = 1000·(n_K_-1)·(1/R2a-1/R1a) and the projections of keratometric astigmatism to the 0°/90° meridian PKc0 = PKast·cos(A1a) and to the 45°/135° meridian PKc45 = PKast·sin(A1a) using a keratometer index of n_K_ = 1.332. The same decomposition was performed on the corneal back surface data (R1p, R2p, A1p): mean back surface power PPmean = 500·(n_A_-n_C_)·(1/R1p+1/R2p), back surface astigmatism PPast = 1000·(n_A_-n_C_)·(1/R2p-1/R1p) and the projections of back surface astigmatism to the 0°/90° meridian PPc0 = PPast·cos(A1p) and to the 45°/135° meridian PPc45 = PPast·sin(A1p) using a refractive index for the cornea n_C_ = 1.376 and the aqueous humour n_A_ = 1.336; and with the TK total corneal power data (R1t, R2t, A1t): mean TK power PTmean = 500·(n_T_-1)·(1/R1t+1/R2t), TK astigmatism PTast = 1000·(n_T_-1)·(1/R2t-1/R1t) and the projections of the TK astigmatism to the 0°/90° meridian PTc0 = PTast·cos(A1t) and to the 45°/135° meridian PTc45 = PTast·sin(A1t) using the refractive index n_T_ = 1.3315 as proposed by Carl-Zeiss-Meditec company for conversion of TK values from mm to dpt and vice versa. The Chang-Waring chord was decomposed into vector components using CWX = CW·cos(CWA) and CWY = CW·sin(CWA) [17,19].

The power vector components in 45°/135° (PKc45, PPc45, PTc45) for keratometry, corneal back surface and total keratometry TK were reversed in sign for all left eyes in our dataset in order to present the data in the same orientation as for right eyes.

### Data processing in Matlab and statistics

For each eye and each parameter (except the axes A1a, A1p, A1t, CWA) the mean and the standard deviation were derived from the sequence of the 3 measurements. The within-subject standard deviation Sw, the repeatability (1.96·$\sqrt{2}$·Sw), and the intra-class correlation ICC were derived from the sequence of 3 repeat measurements for the entire dataset. To assess heteroscedasticity, the standard deviation of the 3 repeat measurements for each eye was analysed as a function of the mean value of the 3 repeat measurements. To investigate the interaction between the uncertainties of the biometric measures, the standard deviation of the 3 repeat measurements were cross-correlated (Spearman correlation coefficient ρ and significance level p based on a first order error of α = 5%) for all parameters (except the axes A1a, A1p, A1t, CWA). Explorative data analysis in tables was performed for the arithmetic mean, the SD, the median, and the lower and upper boundary of the 95% confidence interval (which refers to the 2.5% and 97.5% quantiles). Scatterplots were used to show the standard deviations of the sequence of 3 repeat measurements for the most relevant biometric measures as a function of the mean of the sequence of 3 repeat measurements (together with least squares linear fit lines), and cumulative probability density plots (CDF plots) were used to show the overall distributions of the uncertainties of the biometric measures. Double angle plots were used to display the distributions of the vector components in 0°/90° and 45°/135° for the keratometric astigmatism, back surface astigmatism, and the TK astigmatism. The vector components of the Chang-Waring chord were displayed with their projections to the horizontal (CWX) and vertical (CWY) axis [19].

## Results

From the N = 3379 biometric measurements transferred to us, and after considering the selection criteria, a dataset with N = 2031 measurements (N = 677eyes of 677 patients) was selected for our analysis (327 right and 350 left eyes of 423 female and 254 male patients). From the 3379–2031 = 1348 biometric measurements that were not considered in this study 520 were filtered out as we had measurements of both eyes, 229 were filtered out as we had more than 3 measurements per eye, 286 were filtered out as we had fewer than 3 measurements per eye, and 313 measurements were not considered on the basis of incomplete data or ‘Failed’ / ‘Warning’ messages.

**Table 1A** shows the descriptive data patient characteristics, including Age and the mean of the sequence of 3 repeat measurements with AL, ACD, AQD, LT, CCT, CD, PUP and CW. **Table 1B** displays the explorative data for the keratometry, corneal back surface power, and total keratometry TK in terms of equivalent power (.eq), astigmatism (.ast), and the projections of the astigmatism to the 0°/90° axis (.c0) and to the 45°/135° axis (.c45). All data represent the mean values of the sequence of 3 repeat measurements per eye.

**Table 1:** a: Explorative data of the population characteristics including age, axial length AL, anterior chamber depth measured from corneal epithelium to lens front apex ACD, aqueous depth measured from corneal endothelium to lens front apex AQD, thickness of the crystalline lens LT, central corneal thickness CCT, horizontal corneal diameter CD, pupil size PUP, and Chang-Waring chord CW. The data represent the mean values of a sequence of 3 repeat measurements showing the arithmetic mean value, standard deviation, median, and the lower and upper boundaries of the 95% confidence interval (2.5% and 97.5% quantiles). **b**: Explorative data of the keratometric, corneal back surface, and total keratometry TK corneal power data with mean power (.eq), astigmatism (.ast), and projection of the astigmatism to the 0°/90° axis (.c0) and the 45°/135° axis (.c45). The data represent the mean values of a sequence of 3 repeat measurements showing the arithmetic mean value, standard deviation, median, and the lower and upper boundaries of the 95% confidence interval (2.5% and 97.5% quantiles).

N = 677	Age in years	AL in mm	ACD in mm	AQD in mm	LT in mm	CCT in mm	CD in mm	PUP in mm	CW in mm
Mean	68.5955	24.1765	3.1882	2.6518	4.4089	0.5391	12.0035	3.7997	0.3309
Standard deviation	8.3939	1.6510	0.3848	0.3879	0.4253	0.0339	0.4097	1.0142	0.1887
Median	69.4018	24.0388	3.1732	2.6526	4.4039	0.5397	12.0141	3.7310	0.3041
2.5% quantile	50.7351	21.2350	2.3536	1.7971	3.5858	0.4733	11.1560	2.1705	0.0545
97.5% quantile	84.3012	27.8962	3.9165	3.3794	5.2875	0.6046	12.8002	6.5454	0.7797

N = 677; data in dpt	Keratometry				Corneal back surface				Total keratometry TK			
	PKeq	PKast	PKc0	PKc45	PPeq	PPast	PPc0	PPc45	PTeq	PTast	PTc0	PTc45
Mean	43.1785	0.9810	0.1735	-0.0492	-5.9067	-0.2627	-0.1635	0.0757	43.1099	0.9995	0.0369	0.0182
Standard deviation	1.9153	0.7864	1.0749	0.6059	0.2688	0.1341	0.1724	0.1391	1.9651	0.7876	1.0994	0.6153
Median	43.1855	0.7633	0.1727	-0.0684	-5.8973	-0.2387	-0.1645	0.0746	43.1491	0.7810	0.0244	-0.0041
2.5% quantile	38.8974	0.1329	-2.1764	-1.1435	-6.4506	-0.6085	-0.5120	-0.1793	38.5590	0.1744	-2.4175	-1.0928
97.5% quantile	46.6360	3.2097	2.5388	1.1671	-5.4653	-0.0772	0.1734	0.3493	46.6982	3.2788	2.3889	1.2616

**Fig 1:**
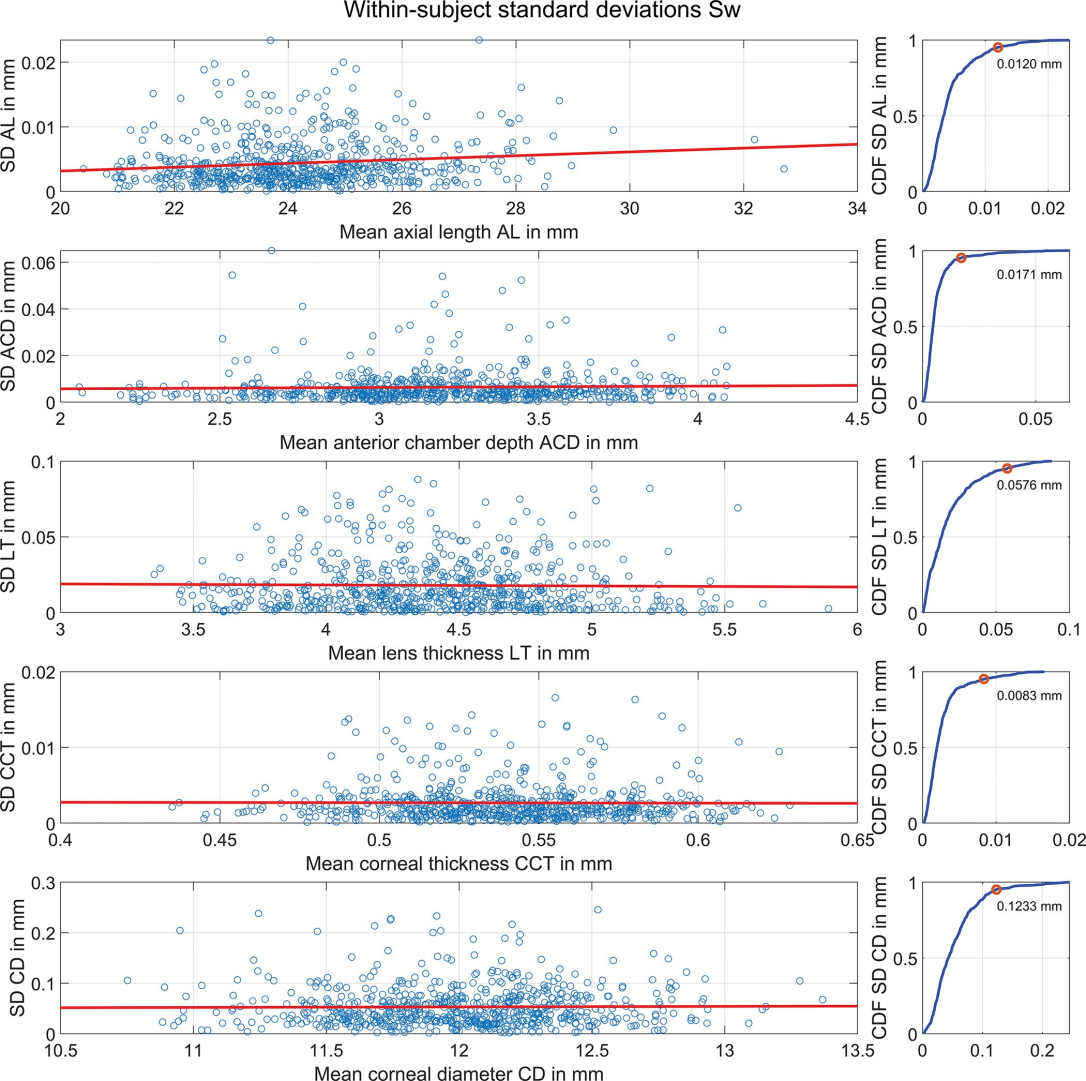
The scatterplots show the standard deviations of the 3 repeat measurements for each eye plotted against the mean values of the 3 repeat measurements, for the most relevant distances used for intraocular lens power calculation together with a least squares linear fit line. AL, ACD, LT, CCT, and CD refer to the axial length, anterior chamber depth measured from corneal epithelium to the lens front apex, thickness of the crystalline lens, central corneal thickness, and horizontal diameter of the cornea. The graphs on the right side show the respective CDF plots of the standard deviations for all eyes; the 95% quantile is marked with a red circle and the respective SD value is provided in the legends. We directly see from the graphs that the standard deviation is mostly constant over the entire parameter range for ACD, LT, CCT and CD, whereas for AL the standard deviation increases systematically for longer eyes.

The scatterplots in **Fig 1** show the standard deviations of the 3 repeat measurements for each eye plotted against the mean values of the 3 repeat measurements for the most relevant distances used for intraocular lens power calculation, together with a least squares linear fit line. The graphs on the right side show the respective CDF plots of the standard deviations for all eyes; the 95% quantile is marked with a red circle and the respective SD value is provided in the legends. We see from the graphs that the standard deviation is mostly constant over the entire parameter range for ACD, LT, CCT and CD, whereas for AL the standard deviation increases systematically for longer eyes. This means that the axial length measurement shows larger uncertainties for myopic eyes compared to hyperopic eyes. In **Table 2** the within-subject standard deviation Sw, the repeatability, the coefficient of variation, and the intra-class coefficient are listed for the sequence of 3 repeat measurements.

**Table 2:** Listing of the within-subject standard deviation Sw, the repeatability, the coefficient of variation, and the intra-class coefficient. AL, ACD, AQD, LT, CCT, CD, PUP, CW refer to the axial length, anterior chamber depth measured from the corneal epithelium to the lens front apex, aqueous depth measured from the corneal endothelium to the lens front apex, thickness of the crystalline lens, central corneal thickness, horizontal corneal diameter, pupil size and Chang-Waring chord. Pxeq, Pxast, Pxc0, and Pxc45 refer to the equivalent power, astigmatic power, and the projections of the astigmatic power to the 0°/90° and 45°/135° axis where x refers to the keratometry (K), corneal back surface power (P), or total keratometry (T), respectively. The CoV data for measures with a mean around zero (CW, (.ast), (.c0), and (.c45)) are also listed.

N = 677	Within-subject standard deviation Sw	Repeatability	Coefficient of variation CoV in %	Intra-class correlation ICC
AL in mm	0.0044	0.0122	0.0182	1.0000
ACD in mm	0.0064	0.0177	0.2030	0.9998
AQD in mm	0.0071	0.0197	0.2726	0.9997
LT in mm	0.0180	0.0499	0.4135	0.9989
CCT in mm	0.0027	0.0074	0.5008	0.9959
CD in mm	0.0532	0.1474	0.4438	0.9912
PUP in mm	0.2235	0.6190	5.8782	0.9642
CW in mm	0.0432	0.1196	-	0.8779
PKeq in dpt	0.0662	0.1833	0.1510	0.9980
PKast in dpt	0.1114	0.3087	-	0.9882
PKc0 in dpt	0.1158	0.3206	-	0.9912
PKc45 in dpt	0.1177	0.3262	-	0.9707
PPeq in dpt	0.0381	0.1055	0.6454	0.9888
PPast in dpt	0.0656	0.1816	-	0.8868
PPc0 in dpt	0.0712	0.1973	-	0.9226
PPc45 in dpt	0.0705	0.1953	-	0.8761
PTeq in dpt	0.0796	0.2204	0.1824	0.9980
PTast in dpt	0.1360	0.3766	-	0.9840
PTc0 in dpt	0.1409	0.3904	-	0.9894
PTc45 in dpt	0.1435	0.3975	-	0.9657

**Table 3** displays the correlations between the standard deviations of the biometric measures derived from the sequence of 3 repeat measurements. The upper right triangular matrix displays the Spearman rank correlation coefficient ρ, and the lower left triangular matrix shows the respective significance level. From the table we can see that the uncertainties of several biometric measures used for lens power calculation are correlated, impeding the use of Gaussian error propagation strategies.

**Table 3:** Correlation between the standard deviations of biometric measures derived from the sequence of 3 repeat measurements. The upper right triangular matrix displays the Spearman rank correlation coefficient, and on the lower left triangular matrix lists the respective significance levels. AL, ACD, AQD, LT, CCT, CD, PUP, CW, PKeq, PPeq, and PTeq refer to the standard deviations of the axial length, anterior chamber depth measured from the corneal epithelium to the lens front apex, aqueous depth measured from the corneal endothelium to the lens front apex, thickness of the crystalline lens, central corneal thickness, horizontal corneal diameter, Pupil diameter, Chang-Waring chord, keratometric equivalent power, equivalent power of the corneal back surface, and equivalent power of total keratometry TL. Statistically significant correlations with a significance level less than 0.05 are marked in bold.

N = 677; correlations of standard deviations		Spearman rank correlation coefficient ρ										
		AL in mm	ACD in mm	AQD in mm	LT in mm	CCT in mm	CD in mm	PUP in mm	CW in mm	PKeq in dpt	PPeq in dpt	PTeq in dpt
Significance level p	AL		**0.1612**	**0.2001**	0.0080	0.3469	0.0613	-0.0252	**0.1082**	0.0603	**0.0920**	**0.0913**
	ACD	**<0.0001**		**0.8393**	**0.1818**	0.2362	0.0007	0.0067	0.0285	0.0350	**0.1308**	**0.0836**
	AQD	**<0.0001**	**<0.0001**		**0.1913**	0.3366	0.0304	0.0244	0.0281	0.0095	**0.1105**	0.0710
	LT	0.8364	**<0.0001**	**<0.0001**		0.1317	0.0230	0.0179	-0.0115	0.0049	**0.0940**	0.0290
	CCT	<0.0001	<0.0001	<0.0001	0.0006		0.0539	-0.0120	0.0425	0.0837	**0.1561**	0.1184
	CD	0.1113	0.9854	0.4291	0.5503	0.1610		0.0373	**0.1118**	0.0221	0.0284	0.0093
	PUP	0.5119	0.8626	0.5261	0.6426	0.7546	0.3323		**0.2569**	0.0052	0.0003	0.0146
	CW	**0.0048**	0.4595	0.4656	0.7658	0.2699	**0.0036**	**<0.0001**		0.0479	0.0221	0.0136
	PKeq	0.1167	0.3636	0.8053	0.8991	**0.0294**	0.5664	0.8930	0.2129		0.1188	0.7567
	PPeq	**0.0167**	**0.0007**	**0.0040**	**0.0145**	**<0.0001**	0.4605	0.9934	0.5661	0.0020	0.0070	0.2593
	PTeq	**0.0175**	**0.0297**	0.0647	0.4514	0.0020	0.8094	0.7045	0.7247	<0.0001	<0.0001	

**Fig 2:**
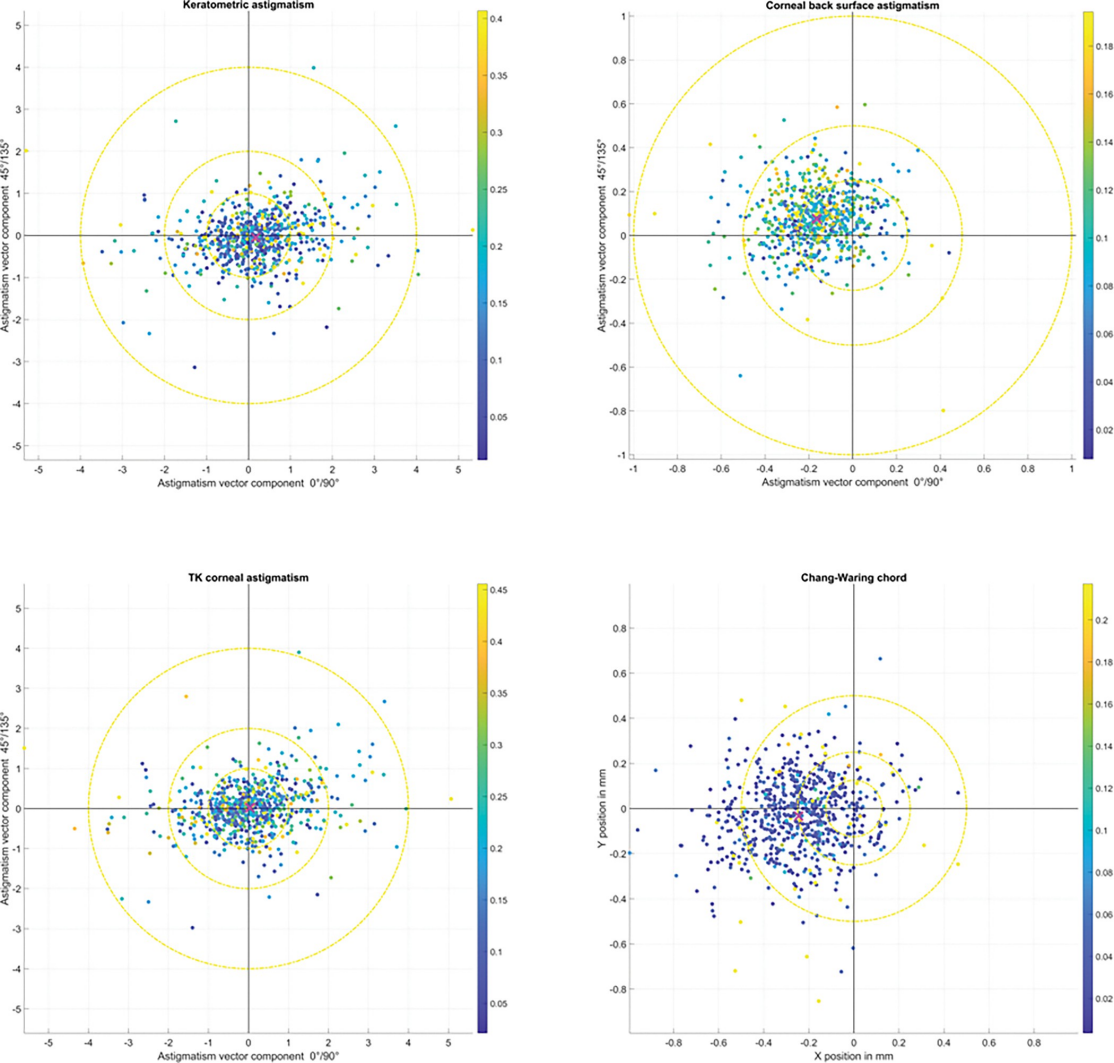
Double angle plots showing the vector components for the keratometric astigmatism (PKc0 and PKc45, upper left graph), for the corneal back surface astigmatism (PPc0 and PPc45, upper right graph), and for the total keratometery TK astigmatism (PTc0 and PTc45, lower left graph). The projections to the 0°/90° and to the 45°/135° axes are displayed on the X and Y axis respectively. The plot on the lower right shows the horizontal and vertical components of the Chang-Waring chord CW as the vector offset between the Purkinje I reflex and the pupil centre. The individual standard deviation of the sequence of 3 measurements is color-coded, and the colour bar on the right of each plot refers to the standard deviation as a measure of uncertainty. For all left eyes the astigmatic vector components with the projections to the 45°/135° axis and the horizontal displacement of the Chang-Waring chord are flipped in sign. The centroid of the scatter is indicated with magenta ‘X’-markers in all plots.

The double angle plots in **Fig 2** show the vector components for the keratometric astigmatism (PKc0 and PKc45, upper left graph), for the corneal back surface astigmatism (PPc0 and PPc45, upper right graph), and for the total keratometry TK astigmatism (PTc0 and PTc45, lower left graph). The projections to the 0°/90° and to the 45°/135° axes are displayed on the X and Y axis respectively. The plot on the lower right shows the horizontal and vertical component of the Chang-Waring chord CW as the vector offset between the Purkinje I reflex and the pupil centre. The individual standard deviation of the sequence of 3 measurements is color-coded, and the colour bar on the right of each plot refers to the standard deviation as a measure of uncertainty. The centroid of the scatter is indicated by magenta ‘X’-markers in all 4 graphs. From the double angle plots we see that the uncertainty of the keratometric, corneal back surface, and TK astigmatism show no clear dependency on the amount or orientation of the astigmatism. From the lower right graph we see that in most of the cases the uncertainty of the Chang-Waring chord is low for repeat measurements, but in some cases randomly distributed over the X-Y plane the Chang-Waring chord shows a large amount of variation for repeat measurements.

## Discussion

Optical biometry is currently used as standard to derive the measures required for lens power calculation. In addition to independence from the examiner, optical biometry is known to provide reliable results, and in contrast to the classical ultrasound biometry all measures are extracted along the visual axis [15,18–25]. All modern IOL power calculation concepts are optimised to yield best results with optical biometry. However, the measures from optical biometers are not fully consistent: With repeat measurements the results may slightly vary [4–14], and the results from different instruments on the market may differ [15,18,20–25].

In general we have to distinguish between deterministic and stochastic errors: deterministic errors in optical biometry could be due to different wavelengths of the light source, differences in the signal processing strategies, the calibration of the instrument, or differences in the location where corneal radius is evaluated or how the radius is interpreted as corneal power. Several papers have been published which investigate the repeatability of biometers [4–14] or comparisons between different biometers on the market [15,18,20–25]. However, the uncertainties of the biometric measures are in themselves not relevant for the surgeon or the patient [3].

The most important issue in context of measurement uncertainties of biometers is the effect of all the uncertainties on the predicted IOL power or the predicted refraction after cataract surgery [1,3]. This means that only those uncertainties which have a direct impact on the result need to be considered. Some classical so-called theoretical-optical formulae such as the SRKT, Hoffer Q or Holladay formula use only the axial length and corneal radius / keratometric power together with a formula constant and the target refraction to predict the lens power. Instead, the Haigis formula considers the phakic anterior chamber depth in addition to axial length and corneal radius. This means that if a clinician calculates IOLs based on the Haigis formula, the uncertainties in corneal curvature, AL, and ACD will affect the result but the uncertainties in LT or CD will not affect the result. Newer formulae in particular tend to take into account additional measures such as LT, CD, CCT or the cornea as a thick lens model (considering measurements of both surfaces), and therefore it is worth studying these additional measures provided by modern biometers which are not considered in the classical formulae.

However, the missing link in most of the papers published on repeatability is the interaction between the uncertainties. Standard techniques of Gaussian error propagation assume that all uncertainties are normally distributed and uncorrelated, but we are aware that this simplification cannot hold in general. If the measures themselves are correlated, we have to expect that the measurement uncertainties will also show some correlation [3]. In addition, Gaussian error propagation assumes that the uncertainties are constant over the entire parameter range, meaning that e.g. for long or short eyes the repeatability of the AL measurement would be the same.

In the present study, we used a large dataset with biometric measurements taken with the IOLMaster 700 from a cataractous population where repeat measurements have been performed. To obtain a clean dataset, we omitted all data with any ‘Failed’ or ‘Warning’ in any of the measures, and only one eye per patient was considered to avoid statistical biases. From this dataset we selected eyes where a sequence of 3 measurements performed at the same day was available.

In the first step we evaluated the sequences of 3 repeat measurements to extract standard metrics such as within-subject standard deviation Sw, repeatability, or intra-class correlations as performed in several previous studies. In the second step we investigated the interactions between the uncertainties of the relevant measurement parameters, which is a pre-condition for a proper error propagation concept. In this context we also evaluated the measurement uncertainties as a function of the parameter itself, to obtain some insight in the heteroscedasticity of the measures. However, as we were restricted to a sequence of 3 repeat measurements for each eye, we could not derive data on the exact distribution of the uncertainties.

We found that the Sw and repeatability of the parameters considered in our study as listed in **Table 2** matches the results of previous studies reasonably well [4–14]. The repeatability of AL, ACD, and AQD is in a range of 12 to 20 μm, whereas the repeatability for LT is around 50 μm and for CCT it is only 7 μm. The uncertainty of CD is in a range of 150 μm, which is nearly 10% of the width of the 95% confidence interval of CD (around 1.6 mm). It is also not surprising that PUP shows a poor repeatability of more than 0.6 mm. Since the visual axis is related to the pupil centre which varies with the pupil size, some of the measurement uncertainties could also be induced by measuring the eye along different reference lines [19]. And measurement of the lens front and back surface (for reading out the ACD or LT) is anyway challenging as the visual axis is typically not perpendicular to both surfaces, and sophisticated concepts (e.g. including lateral scanning) are required to get reliable measurements for the ACD and LT. For non-toric IOLs, the repeatability of the keratometric, corneal back surface, or total keratometry astigmatism might be of minor relevance. However, even the repeatability of the equivalent power for keratometry or total keratometry is surprisingly high at 0.18 dpt and 0.22 dpt. And if we consider that the change in refractive index at the corneal back surface is much lower compared to the front surface, the repeatability of the equivalent power for the corneal back surface with 0.11 dpt is even more surprising.

However, the most relevant finding in this study is that the uncertainties of the biometric measures derived from the sequence of 3 repeat measurements are not uncorrelated, since this precludes the use of Gaussian error propagation [3]. Even if the very high correlation of ρ = 0.84 between ACD and AQD uncertainty does not play a role in IOL power calculation as normally only one of either ACD or AQD is considered in the calculation strategy, we have to be aware that the uncertainties in ACD / AQD correlate to the uncertainty in AL with ρ = 0.16 / 0.20 and with the uncertainty in LT with ρ = 0.18 / 0.19. The high correlations of CCT with AL / ACD / AQD / LT with ρ = 0.35 / 0.24 / 0.34 / 0.13 may play a minor role in IOL power calculation as the impact of CCT on the predicted IOL power or the predicted refraction is rather low. Even if uncertainties in keratometric equivalent power seem to be independent from the uncertainties of all distances in the eye, the uncertainty of the corneal back surface equivalent power might have some interaction with ACD / AQD / and CCT (ρ = 0.13 / 0.11 / 0.16), which acts also on the uncertainty of the TK equivalent power (ρ = 0.12 with CCT). In addition we found that the uncertainty of the AL measurement derived from the sequence of 3 repeat measurements varies with the mean of the 3 AL measurements as shown in the upper graph in **Fig 1**, and this may make the use of Gaussian error propagation inaccurate. We noticed that for longer eyes the variation in the 3 repeat measurements is larger compared to short eyes. For all other distance measurements in the eye the standard deviation of the 3 repeat measurements seems to be constant over the entire parameter range.

However, the present study has some limitations: A) The data presented here are derived from the IOLMaster 700 optical biometer and the results cannot be generalised to other optical biometers. B) as the study was restricted to a sequence of 3 repeat measurements for each eye there is no data on the distribution of the uncertainties for the repeat measurements. This means that for error propagation we still have to assume that the uncertainties are normally distributed. Studies with a larger number of repeat measurements could help to evaluate the distribution of the uncertainties in the future. C) all measurements considered in this study are from a cataractous population with almost no phakic accommodation. The repeatability of biometric measures in a young population with accommodation might be somewhat different as we expect some dynamics, especially in the ACD or AQD and LT. However, our data might be sufficient for deriving an error propagation model to transfer the biometric uncertainties to the predicted IOL power or pseudophakic refraction after cataract surgery.

**In conclusion**, our data resemble the results of studies concerning the repeatability of the IOLMaster 700 as a modern optical biometer. In addition, since we analysed the dependency of the parameter uncertainties in a sequence of repeat measurements on the mean values and the interaction between the uncertainties for all the parameters, our data could be used for generating an enhanced error propagation model which considers the correlations between the measurement uncertainties to predict the effect on the predicted IOL power and refractive outcome for various values of AL.
